# SHAP based feature selection for machine learning prediction of mycoplasma pneumoniae pneumonia with atelectasis in children

**DOI:** 10.1186/s12887-026-06647-3

**Published:** 2026-03-09

**Authors:** Mian Wang, Tengfei Wang, Linli Zhang, Mengsi Li

**Affiliations:** 1 Department of Pediatrics, Wuhan Wuchang Hospital, Wuhan, 430063 China; 2 School of Transportation and Logistics Engineering, Wuhan University of Technology, Wuhan, 430063 China

**Keywords:** Mycoplasma pneumoniae pneumonia, atelectasis, SHAP, Machine learning, Prediction model

## Abstract

**Background:**

Mycoplasma pneumoniae pneumonia (MPP) with atelectasis in children is the most reported complication of MPP in children. It is associated with serious respiratory issues, pulmonary tissue damage and fibrosis, and systemic complications. Present diagnostic methods, which are mainly based on radiographic imaging, have drawbacks related to delayed case detection and radiation exposure. There is a need for a simple, non-invasive approach in the diagnosis. We aimed to develop machine learning (ML) models to identify MPP with atelectasis in children and support early risk stratification.

**Methods:**

We conducted a retrospective study of 508 hospitalized pediatric patients with MPP from July 2022 to June 2024. Baseline clinical data were extracted from the electronic medical record system. Six tree-based ML models—decision tree (DT), random forest (RF), adaptive boosting (ADB), extreme gradient boosting (XGB), bayesian decision tree (BDT), and neural decision tree (NDT)—were developed. The SHapley Additive exPlanations (SHAP) was used to assess model feature importance, and final predictor subsets were determined through SHAP-guided forward feature selection. Models were tuned by Bayesian optimization within a nested five-fold cross-validation framework, and their performance was evaluated in an independent stratified validation cohort using receiver operating characteristic curve (AUROC), accuracy and decision curve analysis (DCA).

**Results:**

SHAP-guided feature selection generated a specific feature set for each model. These features reflect systemic inflammatory burden, febrile status, adaptive immune response, and target-organ injury. In the training cohort, the RF model demonstrated the best performance, achieving an AUROC of 0.91 (95% CI 0.88–0.93). The performance of RF remained stable in the validation cohort, with an AUROC of 0.89 (95% CI 0.83–0.95). The model exhibited strong classification performance across other key metrics, including sensitivity, specificity, and negative predictive value. The results suggest that the model is generalizable with minimal overfitting.

**Conclusion:**

The interpretable RF model helps clinicians identify early cases of MPP that is complicated with atelectasis in children. This stable and interpretable method offers a practical tool for early risk stratification and timely decision-making.

**Supplementary Information:**

The online version contains supplementary material available at 10.1186/s12887-026-06647-3.

## Introduction

Mycoplasma pneumoniae pneumonia (MPP) is a leading cause of community-acquired pneumonia among school-aged children worldwide and remains a major contributor to pediatric hospitalizations [1–4]. In China, MPP has emerged as the predominant respiratory pathogen among children older than five years, with national surveillance showing distinct epidemic cycles every 2–3 years and peak incidence in those aged 6–10 years [5, 6]. MPP carries some serious consequences, such as obstructive bronchitis, bronchiectasis, atelectasis, and myocardial injury, where atelectasis is reported the most, with about 8.1% of the hospitalized cases [7]. MPP patients develop atelectasis as a result of mucus plug–induced airway obstruction and reflects a mechanically reversible pathological process [8, 9]. the clinical course of the MPP-associated atelectasis in children is more serious, with higher fever, increased inflammatory burden, increased hospital stays, and the need for fiberoptic bronchoscopy for obstructive relief compared with those without atelectasis [10–12]. Because these obstructive changes are structurally defined and potentially reversible when detected early, timely identification of children at risk is crucial to prevent pathological progression and to guide escalation of airway clearance therapy, including consideration of bronchoscopic intervention [9, 10, 12].

In routine clinical practice, children hospitalized with MPP who may be at risk for developing atelectasis are initially assessed using clinical evaluation, basic laboratory testing, and chest imaging. However, standard radiography frequently misses small segmental or early lobar collapse, while chest CT—although more sensitive—is not routinely recommended in uncomplicated MPP due to radiation concerns and is reserved for cases with suspected complications or inconclusive findings [13–15]. Microbiological confirmation using PCR or serology is essential for diagnosing MPP but does not identify which children will progress to atelectasis; moreover, PCR performance varies by sampling and timing, and serological methods require paired or delayed sera [16, 17]. Bronchoscopy is both diagnostic and therapeutic for mucus-plug or cast–forming disease, yet it is invasive and unsuitable for early risk triage [18, 19]. Recent Chinese national guidance therefore emphasizes rational imaging, standardized diagnostic pathways, and the need for admission-time, non-invasive risk stratification to identify MPP patients who may benefit from intensified airway clearance or specialist intervention [20]. A growing body of evidence highlights several routinely obtainable clinical indicators that may assist in early prediction of collapse-prone phenotypes in MPP. For MPP with atelectasis specifically, a study identified fever duration, neutrophil ratio (NEU%), platelet count (PLT), alanine aminotransferase (ALT), and lactate dehydrogenase (LDH) as independent contributors to an early nomogram developed using data from the first 24 h of admission to predict which children, despite receiving standardized treatment, would subsequently progress to atelectasis [11]. Closely related mucus-cast disorders such as plastic bronchitis—often arising from mucus obstruction analogous to mechanisms underlying atelectasis—have been associated with markedly elevated LDH, interleukin-8 (IL-8), decreased complement C3, and hypercoagulability (D-dimer), suggesting a shared biology of intense airway inflammation and impaired clearance [18, 21]. Additional admission-time inflammatory markers, including C-reactive protein (CRP), and prealbumin (PAB), have been linked to bronchial mucus plug formation [8]. Simple derived ratios such as the neutrophil-to-lymphocyte ratio (NLR) or CRP-to-NLR ratio (C-NLR) have also shown utility in stratifying refractory MPP and predicting prolonged fever [22]. Evidence from Chinese cohorts supports LDH—often with ferritin—as a indicator of inflammatory severity and treatment intensity in MPP [23, 24]. Collectively, these routinely measurable biomarkers provide biologically plausible and cost-effective parameters that can inform early identification of children at risk for atelectasis and offer a practical foundation for subsequent ML based predictive modeling.

Most existing pediatric prediction models for MPP–related disorders focus on refractory or severe phenotypes (RMPP/SMPP) or complications such as plastic bronchitis, rather than directly identifying atelectasis risk within the broader MPP population. These models are typically using logistic regression nomograms [19, 25–28]. Recent ML attempts have introduced explainability but still target RMPP/SMPP rather than collapse-prone disease [29, 30]. To date, only one risk-association model specifically addresses MPP-associated atelectasis [11], while another pertains to long-term atelectasis following childhood pneumonia but not early risk stratification for hospitalized MPP [12]. Existing models also frequently rely on variables not universally available before bronchoscopy, lack external validation, and provide limited clinician-interpretable outputs, challenging adherence to TRIPOD principles for transparent prediction-model reporting [31]. To address these gaps, we propose an interpretable, admission-time risk-stratification model for atelectasis in hospitalized children with MPP, leveraging tree-based learners to capture non-linearities and feature interactions while retaining concise clinical signatures. We will incorporate SHAP-based explanations to deliver transparent, clinician-facing interpretability at both model and individual-patient levels, and follow contemporary best-practice guidelines for prediction-model development and reporting (TRIPOD + AI) [32, 33]. Our objective is to create and internally validate an explainable tool using routinely available admission variables to accurately identify MPP patients at risk of atelectasis at presentation.

## Methods

### Ethics approval

This study was approved by the Ethics Committee of Wuhan Wuchang Hospital. As it was a retrospective study, the requirement for informed consent was waived.

### Data extraction

#### Data extraction and study population

This retrospective study included 847 pediatric patients diagnosed with Mycoplasma pneumoniae pneumonia (MPP) who underwent chest computed tomography (CT) examinations at the Department of Pediatrics, Wuhan Wuchang Hospital, between July 2022 and June 2024. After applying inclusion and exclusion criteria and removing extreme or abnormal values, 508 patients were included in the final analysis.

#### Temporal relationship between predictors and outcome

To ensure the predictive validity of the model, we strictly defined the timing of data collection. All candidate predictor variables (demographics, vital signs, and laboratory markers) were collected at the time of hospital admission (baseline), specifically within the first 24 h of presentation. These data represented the patient’s status prior to any escalation of therapy (e.g., corticosteroids or bronchoscopy). The outcome of atelectasis was determined based on radiological confirmation via chest CT performed either upon admission or during hospitalization. This design ensures that the predictors were always measured prior to or simultaneously with the clinical assessment of atelectasis, making the tool suitable for early risk stratification at the point of care.

#### Diagnostic criteria

The diagnosis of MPP followed the Guidelines for the Diagnosis and Treatment of Mycoplasma Pneumonia in Children (2023 Edition). Children were considered to have MPP if they met any one of the following laboratory criteria: (1) A fourfold or greater increase in specific serum IgG antibody titers between the acute and convalescent phases; (2) A specific serum IgM antibody titer ≥ 1:160; (3) A positive PCR test for Mycoplasma pneumoniae DNA or RNA. Patients meeting any one of these three criteria together with clinical manifestations and/or radiological evidence of pneumonia were diagnosed with MPP. A diagnosis of MPP with atelectasis required fulfillment of the MPP diagnostic criteria described above plus radiological confirmation of segmental or lobar atelectasis [34].

#### Exclusion criteria

Patients were excluded if they met any of the following conditions: (1) Prolonged disease course with duration > 14 days at admission; (2) Coinfection with bacteria, viruses, or Mycobacterium tuberculosis; (3) Presence of bronchiectasis, immotile cilia syndrome, congenital immunodeficiency, or hematologic diseases; (4) Presence of immunodeficiency or chronic lung disease; (5) Coexisting renal, hepatic, cardiovascular, or connective tissue diseases; (6) Missing critical data required for study inclusion, specifically patients without a confirmed diagnosis of atelectasis outcome (via chest CT) or essential baseline demographic information. Note: Patients with missing values in specific laboratory variables were retained, and these gaps were addressed via imputation as described below.

#### Incidence rate consideration

It is important to note that because our inclusion criteria required patients to have undergone a chest CT scan—a procedure typically reserved for patients with severe symptoms or suspected complications—our cohort represents a high-risk subgroup of MPP patients. Consequently, the incidence of atelectasis in this study (41.5%) is higher than that reported in general MPP populations (6.9%–8.1%). This selection bias means our model is specifically optimized for risk stratification in clinically severe or complicated cases where advanced imaging is indicated.

### Data pre-processing

To obtain the integrity of the dataset, variables with missing values over 30% were excluded. The remaining variables with missing data were imputed using multiple imputation through the miceforest package in Python [35]. First, variables with a missingness rate exceeding 30% across the entire cohort were excluded from candidate predictors. We assumed that the data were missing at random (MAR); this assumption is biologically reasonable as the predictors (e.g., CRP, SAA, ferritin, CK-MB) are routine clinical laboratory indicators. In our retrospective data extraction, missing values for these variables were primarily attributable to technical factors—such as insufficient sample volume, instrument calibration errors, or sporadic clerical errors—rather than the severity of the atelectasis itself (which would constitute missing not at random, MNAR). Therefore, it is reasonable to assume that the probability of missingness is unrelated to the unobserved data (atelectasis status), satisfying the MAR condition. The dataset was then randomly split into a training set (80%) and a validation set (20%). The validation set was kept completely independent to ensure robust model evaluation. To eliminate scale discrepancies among predictors, feature standardization was applied based on the training set parameters, and the same scaling factors were subsequently applied to the validation set for consistency.

### Statistical analysis

A total of 20 variables were initially selected as candidate predictors. Univariable analyses were performed to assess their correlation with atelectasis. For continuous variables, normality was verified using the Kolmogorov–Smirnov test. For normally distributed data, the comparison was made using the t-test and is reported as mean ± SD. For non-normally distributed data, the Mann–Whitney U test was utilized, and results are expressed as median (IQR). Categorical variables were analyzed using the χ² test or Fisher’s exact test. A two-sided P-value of < 0.05 was considered statistically significant; variables that met this criterion were included in the model construction. All descriptive and univariable statistical analyses were performed in IBM SPSS Statistics, version 27.0 (IBM Corp., Armonk, NY, USA). Python (version 3.11; Python Software Foundation) was used for data imputation and machine-learning model development.

### Feature selection and model construction

To balance model performance and clinical interpretability, a two-stage feature selection process was applied. In the first stage, a pre-model was built for each machine-learning algorithm, including all variables that remained significant in the univariable analysis. For each pre-model, Shapley Additive Explanations (SHAP) were calculated to determine the contribution of each feature to the model output. The mean absolute SHAP value across all samples served as a metric for the global feature importance score, and the features were ranked accordingly [36, 37]. In the second stage, forward feature selection was conducted by starting with the most important feature based on the SHAP ranking, then sequentially adding features based on their ranked priority [38]. Each addition was evaluated using five-fold cross-validation to achieve a best AUROC score. If the addition of a feature improved the AUROC, it was retained; otherwise, it was discarded, and the selection process discontinued. This iterative process continued until no further improvement was observed. The procedure was applied to decide the final subset for each machine learning algorithm.

Six tree-based machine learning algorithms were used to construct prediction models: decision tree (DT), random forest (RF), adaptive boosting (ADB), extreme gradient boosting (XGB), bayesian decision tree (BDT), and neural decision tree (NDT). DT has transparent and rule-based decisions process that enhance clinical interpretability. RF is based on a collection of decorrelated trees, which reduces model variance and is applicable to mid-scale cohort studies in the medical field [39, 40]. ADB sequentially emphasizes misclassified samples, allowing the model to recognize difficult-to-identify patient subgroups [41, 42]. XGB extends boosting to include second derivative and explicit regularization terms, which improves model prediction while reducing overfitting [43, 44]. BDT uses a Bayesian prior on tree structure, which allows inference of posterior parameters, and helps produce stable models in small samples [45, 46]. NDT accommodates a neural network-based representation learning in conjuncture with a tree-based decision boundary, resulting in a model that inherently recognizes complex nonlinear interactions of clinical features [47]. We utilized Bayesian optimization for hyperparameter tuning across all algorithms: hyperparameter sets were iteratively examined to maximize AUROC [48, 49]. Model development followed a nested cross-validation design: the inner cross-validation loop optimized hyperparameters, and the outer loop provided an unbiased assessment of predictive performance and mitigated overfitting. The optimal hyperparameters for each algorithm were then used to retrain the final model on the entire training set, and performance was subsequently evaluated on the validation cohort.

### Model evaluation

Model evaluation was performed on the training cohort and validation cohort respectively. Evaluation metrics included accuracy (the proportion of true results), precision/positive predictive value (PPV; the ratio of correctly predicted instances), recall/sensitivity (the ratio of correctly predicted positive measurements), F1 score (the weighted average between precision and recall), area under the receiver operating characteristic curve (AUROC; the discrimination ability between class labels), specificity (the proportion of true negative observations), and negative predictive value (NPV; the proportion of correctly predicted negative instances) [50]. Each final model was applied to the independent validation set to obtain predictions and calculate these metrics. In this study, AUROC was used as the primary indicator of discriminative ability because it captures the model’s ability to distinguish between positive and negative cases across all possible decision thresholds and is less sensitive to small differences in class proportions, whereas accuracy was used as a secondary comparison metric to summarize overall correctness in our relatively balanced setting. Decision curve analysis (DCA) was conducted to evaluate the clinical utility of the best model by quantifying the net benefit across a range of threshold probabilities. DCA examines whether the model has practical implications compared to treating all or none: therefore, add an extra layer of information for clinical applicability on top of classic benchmarking parameters.

Calibration, reflecting the agreement between predicted probabilities and observed outcomes, was assessed to evaluate the reliability of the model’s risk estimates. For the best-performing Random Forest model, we calculated the Brier score (ranging from 0 to 1, with lower values indicating better accuracy) and generated calibration curves using the validation cohort. The calibration curve plots predicted probabilities against observed frequencies, enabling visual assessment of model calibration across the spectrum of risk scores.

## Results

### Patient characteristics

There were 508 cases in the final cohort, including 297 MPP without atelectasis and 211 MPP with atelectasis. There was no significant difference between the two groups in terms of age or gender distribution. The MPP with atelectasis had a longer fever duration (6 days versus 4 days, *P* < 0.001) and higher peak body temperature (39.0 °C versus 38.5 °C, *P* < 0.001) compared with the MPP without atelectasis group. Furthermore, NEU (both count and %) were higher but LYM lower in the MPP with atelectasis (all *P* < 0.001). Inflammatory and tissue injury markers (SAA, CRP, hs-CRP, LDH, ferritin, IL-6, and PCT) were higher in the MPP with atelectasis group (all *P* < 0.001). Additionally, PLT differed between groups (*P* < 0.001), whereas total WBC, ALT, AST, and IgE levels were not significantly different (Table 1).

**Table 1:** Baseline characteristics of children MPP with and without atelectasis

Variables	MPP without atelectasis (*n* = 297)	MPP with atelectasis (*n* = 211)	*P* value
Sex			0.063
female	133 (44.8%)	113 (53.6%)	
male	164 (55.2%)	98 (46.4%)	
age	7.60 (5.50, 9.70)	8.00 (6.30, 9.50)	0.203
Fever durations	4.00 (0.00, 6.00)	6.00 (4.00, 7.00)	< 0.001
Peak temperature (°C)	38.50 (36.80, 39.00)	39.00 (38.70, 39.50)	< 0.001
WBC (×10⁹/L)	6.83 (5.51, 8.12)	6.92 (5.67, 8.50)	0.258
NEU (×10⁹/L)	3.89 (3.00, 5.05)	4.41 (3.48, 5.75)	< 0.001
LYM (×10⁹/L)	1.96 (1.58, 2.52)	1.70 (1.34, 2.10)	< 0.001
NEU%	0.60 (0.51, 0.66)	0.66 (0.58, 0.72)	< 0.001
SAA (mg/L)	31.35 (10.43, 64.01)	99.44 (57.03, 160.14)	< 0.001
CRP (mg/L)	4.45 (2.10, 7.61)	12.92 (8.43, 24.34)	< 0.001
hs-CRP (mg/L)	6.45 (2.08, 10.75)	18.48 (11.32, 30.54)	< 0.001
PLT (×10⁹/L)	250.00 (206.00, 295.00)	227.00 (189.50, 263.50)	< 0.001
CK-MB (U/L)	3.30 (2.70, 4.10)	2.60 (1.90, 3.45)	< 0.001
LDH (U/L)	247.50 (215.80, 272.10)	251.50 (223.45, 284.50)	0.012
ALT (U/L)	12.60 (10.20, 16.10)	12.40 (9.90, 16.60)	0.509
AST (U/L)	29.60 (24.70, 34.80)	30.00 (25.75, 34.55)	0.548
Ferr (ng/mL)	90.31 (69.30, 118.12)	121.99 (93.25, 160.35)	< 0.001
IL-6 (pg/mL)	12.86 (7.31, 20.22)	18.02 (11.04, 26.14)	< 0.001
PCT (ng/mL)	0.06 (0.04, 0.09)	0.08 (0.05, 0.14)	< 0.001
IgE (IU/mL)	81.00 (32.70, 239.88)	88.48 (35.00, 261.24)	0.324

Continuous variables are expressed as median (interquartile range), and categorical variables as number (percentage). Comparisons between groups were conducted using the Mann–Whitney U test for continuous variables and the chi-square test or Fisher’s exact test for categorical variables

*Abbreviations*: *WBC *White blood cell count, *NEU *Neutrophil count, *LYM *Lymphocyte count, *NEU% *Neutrophil percentage, *SAA *Serum amyloid A, *CRP *C-reactive protein, *h-CRP *high-sensitivity C-reactive protein, *PLT *Platelet count, *CK-MB *Creatine kinase-MB, *LDH *Lactate dehydrogenase, *ALT *Alanine aminotransferase, *AST *Aspartate aminotransferase, *Ferr *Ferritin, *IL-6 *Interleukin-6, *PCT *Procalcitonin, *IgE *Immunoglobulin E

### Identification of important predictive features (Using SHAP Values)

**Fig. 1:**
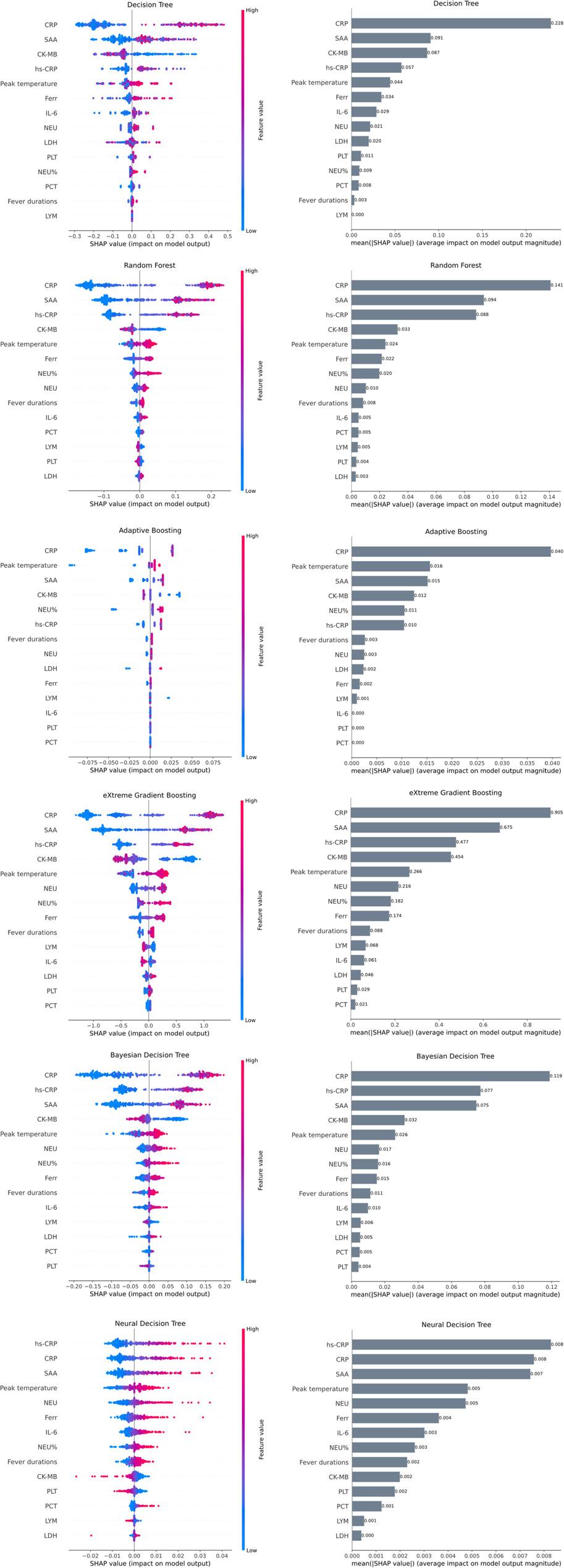
SHAP-based feature importance across machine learning models for predicting MPP. Note: SHAP (Shapley Additive Explanations) results for six models: decision tree (DT), random forest (RF), adaptive boosting (ADB), extreme gradient boosting (XGB), bayesian decision tree (BDT), and neural decision tree (NDT). For each model, the right is a bar plot of the mean absolute SHAP value for each variable, which reflects how much that variable contributes to the model on average. The left is the SHAP summary dot plot for the same variables. Each dot represents one patient; the position on the x-axis is the SHAP value (positive values push the prediction toward MPP, negative values away from MPP), and the color shows the original value of the variable (blue = lower, pink = higher)

All 14 variables that yielded statistically significant results during univariate analysis were presumed as candidate predictors for all machine learning models in the beginning. For each model, the importance of the features was determined using Shapley Additive Explanations (SHAP). In this manner, SHAP summary dot plots and mean absolute SHAP value bar plots depicted the direction and magnitude of each variable’s effect on the model’s MPP with atelectasis prediction probability (Fig. 1). This facilitates a convenient method of interpreting the clinical and laboratory features more intuitively that can change the outcome from the model by a positive or negative way.

Accordingly, we ranked the predictors identified using a SHAP-derived importance ranking and utilized a forward stepwise feature addition strategy to optimize each model. Starting with the top-ranked predictor, other features were sequentially added in descending SHAP importance until improving the models no longer enhanced training set performance. This process was repeated for all six models (DT, RF, ADB, XGB, BDT, and NDT), with the respective features subsequently used for training and evaluating final models. We inferred the final subset of predictors for each algorithm (Table 2).

**Table 2:** Final Predictors selected by SHAP-Based feature selection and forward stepwise optimization

Model	Predictors
DT	CRP, SAA, CK-MB, hs-CRP, Peak temperature, Ferr, IL-6
RF	CRP, SAA, hs-CRP, CK-MB, Peak temperature, Ferr
ADB	CRP, Peak temperature, SAA, CK-MB, NEU%, hs-CRP
XGB	CRP, SAA, hs-CRP, CK-MB, Peak temperature, NEU, NEU%, Ferr, Fever durations, LYM
BDT	CRP, hs-CRP, SAA, CK-MB, Peak temperature
NDT	hs-CRP, CRP, SAA, Peak temperature, NEU, Ferr, IL-6, NEU%, Fever durations, CK-MB, PLT

Predictors were selected based on the feature importance ranking obtained from SHAP (Shapley Additive Explanations) analysis, followed by a forward stepwise feature-addition procedure to maximize AUROC. All variable names correspond to clinical and laboratory parameters used for model training

### Sensitivity analysis for missing data

To evaluate the robustness of our imputation strategy and assess potential impacts of missing data, we conducted a sensitivity analysis comparing models trained on imputed datasets with those trained using complete case analysis (i.e., excluding all patients with any missing predictor values) (Supplement Fig. 1). The AUROC curves of models trained on imputed datasets were higher than those of non-imputed models, though the differences were not statistically significant. The consistency in performance across these different approaches suggests that our multiple imputation method is valid and that the reported model performance is not merely an artifact of the data imputation technique.

### Performance of machine learning models

Following the SHAP-based feature selection and forward stepwise optimization, the final predictor subsets were used to train and evaluate the six machine-learning algorithms. In the training cohort, all models achieved good discrimination, with AUROC values ranging from 0.83 for DT to 0.91 for RF (Table 3). Ensemble methods (RF, ADB, and XGB) as well as BDT and NDT all yielded AUROC between 0.88 and 0.91, together with accuracies of 0.80–0.83. Precision/PPV, recall/sensitivity, F1 score, specificity, and NPV across models suggest that the optimized feature sets allowed robust learning without overfitting.

**Table 3:** Performance of machine learning models in the training set

Model	Accuracy	Precision / PPV	Recall / Sensitivity	F1 Score	AUROC	Specificity	NPV
DT	0.76 (0.75, 0.77)	0.72 (0.68, 0.77)	0.70 (0.64, 0.77)	0.71 (0.69, 0.72)	0.83 (0.79, 0.87)	0.80 (0.74, 0.86)	0.79 (0.77, 0.82)
RF	0.83 (0.81, 0.85)	0.81 (0.76, 0.84)	0.79 (0.77, 0.82)	0.80 (0.78, 0.82)	0.91 (0.88, 0.93)	0.86 (0.82, 0.90)	0.85 (0.84, 0.87)
ADB	0.83 (0.78, 0.87)	0.80 (0.73, 0.86)	0.80 (0.76, 0.85)	0.80 (0.75, 0.84)	0.90 (0.87, 0.93)	0.85 (0.79, 0.91)	0.86 (0.83, 0.89)
XGB	0.80 (0.77, 0.82)	0.73 (0.70, 0.78)	0.82 (0.79, 0.85)	0.77 (0.75, 0.79)	0.89 (0.86, 0.92)	0.78 (0.74, 0.84)	0.86 (0.84, 0.88)
BDT	0.81 (0.79, 0.84)	0.77 (0.72, 0.84)	0.78 (0.76, 0.81)	0.78 (0.75, 0.80)	0.89 (0.86, 0.92)	0.83 (0.78, 0.89)	0.84 (0.83, 0.85)
NDT	0.81 (0.77, 0.83)	0.77 (0.72, 0.80)	0.78 (0.72, 0.82)	0.77 (0.72, 0.80)	0.88 (0.85, 0.91)	0.84 (0.80, 0.86)	0.84 (0.80, 0.86)

Model performance was evaluated using a nested five-fold cross-validation strategy on the training dataset. Values are presented as the mean and 95% confidence intervals obtained across the outer validation folds. decision tree (DT), random forest (RF), adaptive boosting (ADB), extreme gradient boosting (XGB), bayesian decision tree (BDT), and neural decision tree (NDT). Metrics include Accuracy, Precision / Positive Predictive Value (PPV), Recall / Sensitivity, F1 Score, AUC(ROC), Specificity, and Negative Predictive Value (NPV)

**Fig. 2:**
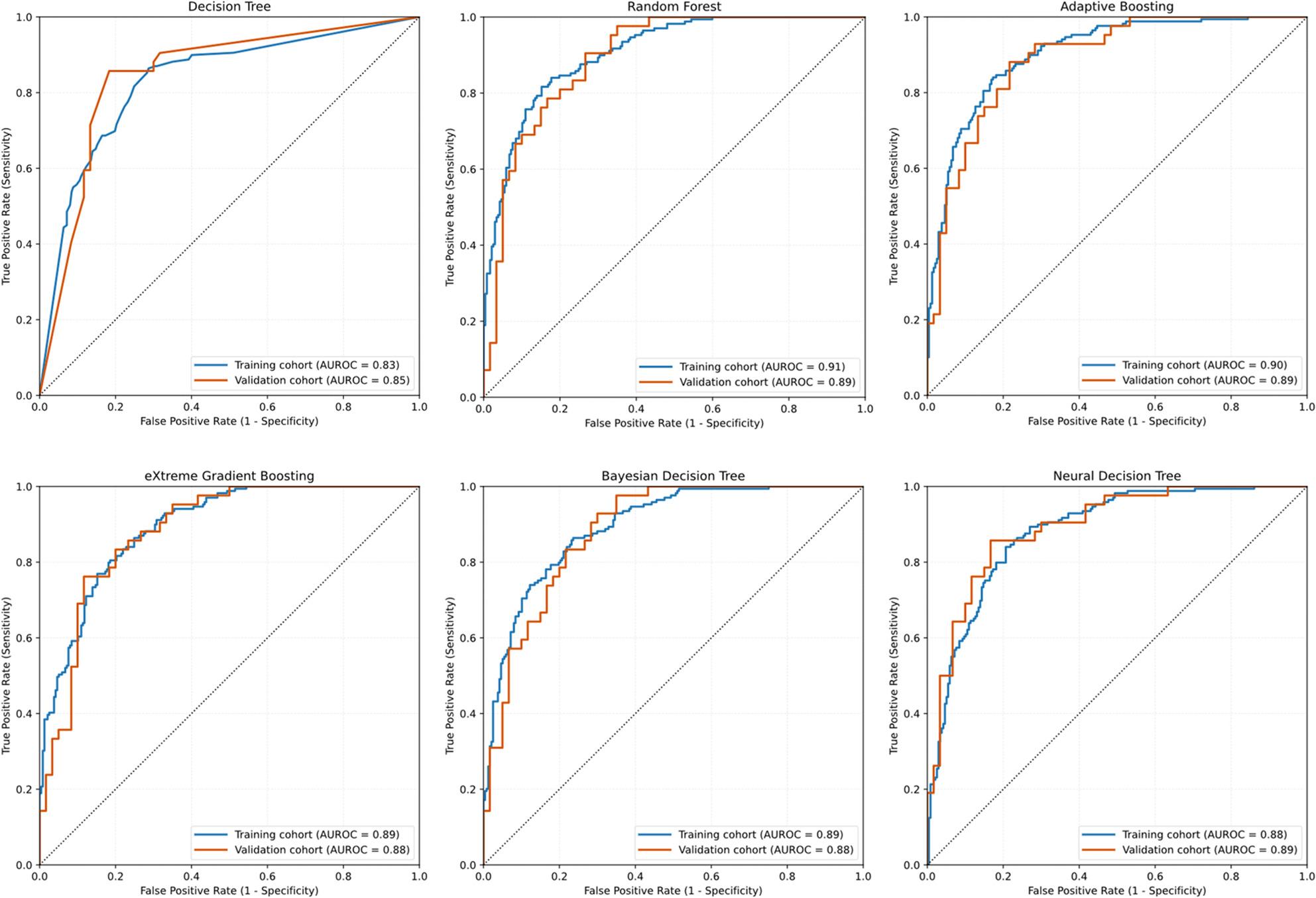
Receiver Operating Characteristic (ROC) Curves of Machine Learning Models for Predicting Pediatric Mycoplasma pneumoniae Pneumonia with atelectasis. Note: AUROC curves are shown for the training and validation cohorts across the six algorithms. Each panel displays the model-specific AUROC for both cohorts, illustrating overall discriminative performance and the degree of generalizability. The close alignment between the blue (training) and orange (validation) curves indicates stable model performance with minimal overfitting

Model performance remained strong in the validation cohort (Table 4). AUROC values ranged from 0.85 (DT) to 0.89 for RF, ADB, and NDT, with intermediate performance for XGB and BDT (AUROC 0.88). Across these models, accuracy was approximately 0.80–0.83, with balanced precision (0.74–0.80), recall (0.72–0.79), and F1 scores (0.75–0.78). Specificity and NPV also remained high (0.80–0.87 and 0.81–0.85, respectively). AUROC (Fig. 2) showed close concordance between the training and validation cohorts across all six algorithm models, providing good generalizability of the optimized models for predicting pediatric MPP with atelectasis.

**Table 4:** Performance of machine learning models in the validation set

Model	Accuracy	Precision / PPV	Recall / Sensitivity	F1 Score	AUROC	Specificity	NPV
DT	0.80 (0.73, 0.87)	0.79 (0.65, 0.91)	0.72 (0.57, 0.84)	0.75 (0.63, 0.85)	0.85 (0.76, 0.92)	0.87 (0.78, 0.94)	0.81 (0.72, 0.90)
RF	0.81 (0.74, 0.88)	0.77 (0.64, 0.89)	0.79 (0.65, 0.90)	0.78 (0.67, 0.86)	0.89 (0.83, 0.95)	0.83 (0.74, 0.93)	0.85 (0.75, 0.93)
ADB	0.82 (0.75, 0.88)	0.78 (0.65, 0.91)	0.77 (0.64, 0.89)	0.77 (0.67, 0.86)	0.89 (0.82, 0.95)	0.85 (0.75, 0.94)	0.84 (0.75, 0.92)
XGB	0.80 (0.72, 0.87)	0.74 (0.61, 0.86)	0.79 (0.65, 0.91)	0.76 (0.65, 0.85)	0.88 (0.82, 0.94)	0.80 (0.70, 0.90)	0.84 (0.75, 0.93)
BDT	0.80 (0.72, 0.87)	0.75 (0.61, 0.87)	0.77 (0.63, 0.88)	0.75 (0.64, 0.85)	0.88 (0.82, 0.94)	0.82 (0.72, 0.91)	0.83 (0.73, 0.92)
NDT	0.83 (0.75, 0.89)	0.80 (0.68, 0.92)	0.76 (0.63, 0.89)	0.78 (0.68, 0.87)	0.89 (0.83, 0.95)	0.87 (0.78, 0.95)	0.84 (0.74, 0.93)

Values are presented as point estimates with 95% confidence intervals derived from the independent validation dataset. Model abbreviations are identical to those in Table 3

#### Calibration of the random forest model

**Fig. 3:**
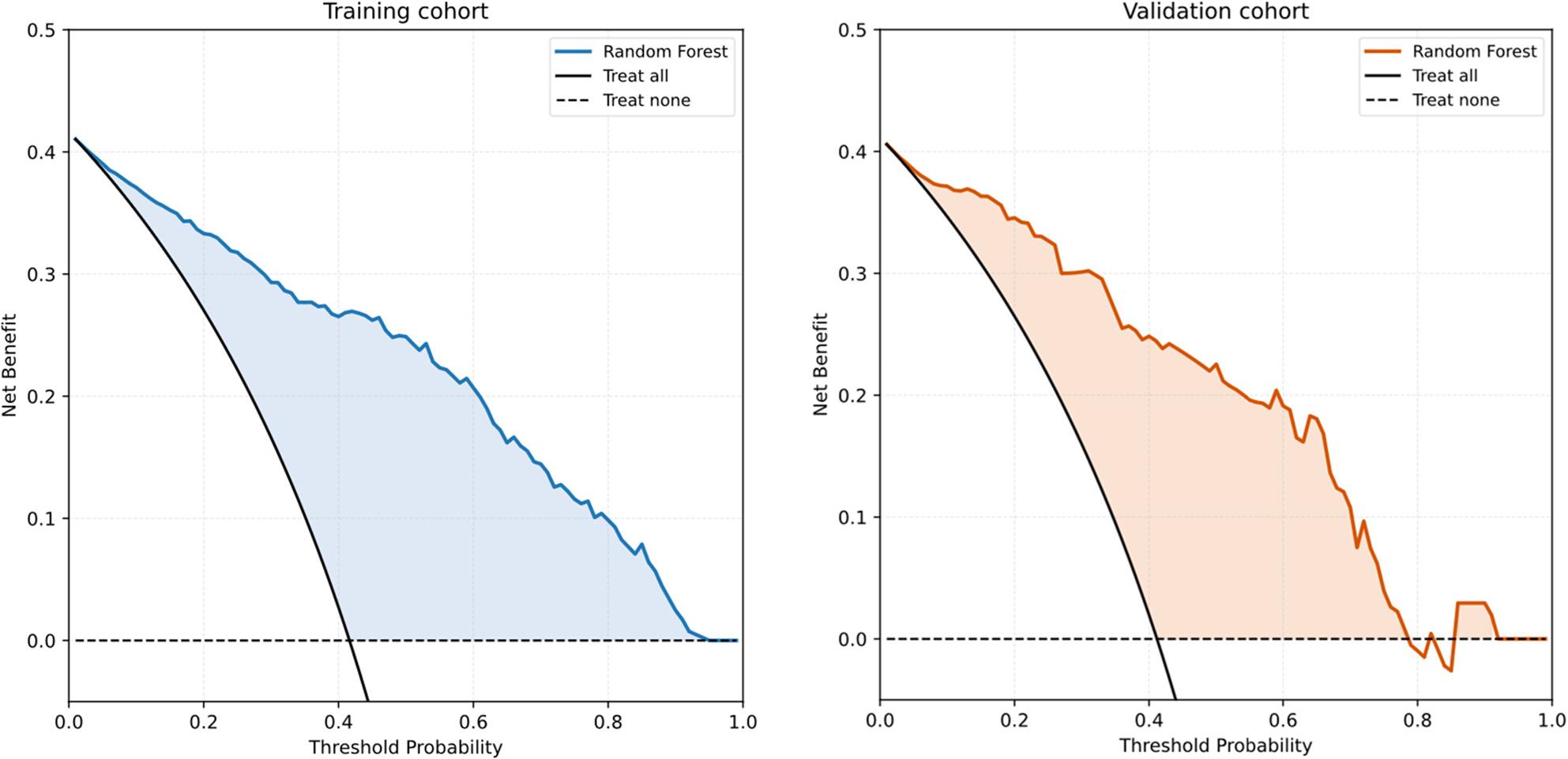
Decision Curve Analysis of the Random Forest Model in the Training and Validation Cohorts. Note: Decision curve analysis (DCA) for the Random Forest model demonstrates its net clinical benefit across a continuum of threshold probabilities in both the training (left panel) and validation (right panel) cohorts. The RF curve is compared against the treat all and treat none reference strategies. Across most threshold ranges, the RF model yields higher net benefit, indicating its potential utility for individualized risk-based decision-making in pediatric MPP with atelectasis

Assessment of calibration revealed that the Random Forest model provided reliable probability estimates. The Brier score for the validation cohort was 0.023, indicating a relatively small mean squared error between the predicted probabilities and actual outcomes. The calibration curve (Supplement Fig. 2) demonstrated close alignment with the ideal diagonal line, particularly in the intermediate-to-high risk ranges, suggesting that the model’s predicted probabilities are well-calibrated and can be trusted for clinical decision-making.Decision curve analysis was performed for the RF model to assess its potential clinical utility (Fig. 3). In both the training and validation cohorts, RF consistently provided greater net benefit than the “treat all” and “treat none” strategies across a wide range of clinically relevant threshold probabilities. Taken together, these findings indicate that, while several algorithms demonstrated comparably high discrimination, the RF model offered the most favorable combination of predictive performance and clinical net benefit and therefore represents the most promising tool for risk stratification in this setting.

## Discussion

In this retrospective cohort study, we successfully developed and validated a SHAP-guided Random Forest (RF) classifier for the early identification of atelectasis risk in hospitalized children with MPP using routine admission variables. Among the six tree-based models evaluated, the RF classifier demonstrated the most optimal trade-off between discrimination and stability, achieving an AUROC of 0.89 in the independent validation cohort with minimal evidence of overfitting. The final predictor subset—including CRP, hs-CRP, SAA, CK-MB, peak temperature, and ferritin—forms a biologically plausible “inflammation-and-injury” signature. Pathophysiologically, the combination of prolonged fever duration, elevated peak temperature, and neutrophil dominance reflects a sustained cytokine storm and innate immune-driven hypersecretion, which are known precursors to mucus plug formation and lobar collapse. Concurrently, elevated acute-phase reactants (CRP, SAA) and ferritin serve as indicators of systemic inflammation and tissue injury, respectively. This consistency between the model’s selected features and the underlying pathophysiology of MPP-associated atelectasis reinforces the clinical credibility of the algorithm.

Compared to existing literature, which has predominantly focused on refractory MPP or plastic bronchitis using traditional regression-based nomograms, this study introduces significant methodological innovations [11, 21, 27, 32]. Pediatric clinical data are inherently characterized by non-linear relationships and high-order interactions that linear models often fail to capture. The RF algorithm, benefiting from ensemble averaging across de-correlated trees, effectively models these complex structures without imposing strict distributional assumptions. Furthermore, our adherence to the TRIPOD-AI guidelines—utilizing nested cross-validation for hyperparameter optimization and maintaining a strict separation between training and independent validation sets—addresses the common pitfalls of “optimism bias” seen in single-center studies. While the two-stage SHAP-guided feature selection introduces higher computational complexity than traditional univariate filtering or Lasso regression, our analysis demonstrates that this complexity is necessary and justified. It captures critical interactions that simpler methods miss, as evidenced by the high concordance between the training (AUROC 0.91) and validation (AUROC 0.89) cohorts, confirming that the model complexity did not compromise generalizability.

Recent comparative analyses suggest that machine learning (ML) models often do not significantly outperform conventional logistic regression (LR) in predicting clinical outcomes using structured tabular data. Our findings align with this observation; although our optimized Random Forest model achieved a slightly higher AUROC (0.89) compared to a benchmark LR model (0.84), the performance differential was marginal (Supplement Table 1, Supplement Fig. 3). However, the rationale for employing a tree-based ML approach extends beyond discrimination metrics. The pathogenesis of pediatric MPP involves complex, non-linear interactions between systemic inflammatory markers (e.g., CRP, SAA) and end-organ injury markers (e.g., Ferritin, CK-MB). The ML framework, coupled with SHAP explanations, automatically quantifies these high-order interactions without the need for pre-specifying interaction terms—a task often challenging for traditional LR models. Moreover, regarding clinical usability, the SHAP-based interpretability offers patient-level decision support that mirrors clinicians’ reasoning processes, providing a transparent view of individual biomarker contributions. Thus, we propose that the value of this model lies not in replacing LR, but in offering a biologically plausible and interpretable tool for risk stratification in complex inflammatory conditions.

Beyond robust discrimination, the principal contribution of this study lies in the enhanced interpretability afforded by the SHAP framework. Unlike conventional “black-box” ML approaches, the integration of SHAP values transforms complex model outputs into transparent, evidence-based insights. This interpretability aligns the model’s feature importance—such as the combined signature of CRP and ferritin—with established pathophysiology, thereby bridging the gap between algorithmic performance and clinical reasoning. By providing a tool that is both powerful and explainable, we address a critical barrier to the adoption of AI in clinical practice, ensuring the model serves as a supportive decision aid rather than an opaque arbiter.

Translating these promising results into clinical practice requires a pragmatic implementation strategy that bridges the gap between algorithmic performance and bedside decision-making [51]. To prevent “alert 4” and ensure the tool acts as an aid rather than an interruption, we propose a tiered risk-stratified protocol integrated into the Electronic Health Record (EHR) workflow [50]. Depending on the clinical context—whether the priority is to rule out disease (maximizing sensitivity) or to justify invasive intervention (maximizing specificity)—the probability threshold can be adjusted. For routine deployment, we recommend a three-tiered system: Low Risk triggers standard care; Intermediate Risk prompts intensified physiotherapy and expedited imaging; and High Risk activates alerts for anesthesia and respiratory teams to prepare for bronchoscopy. This approach ensures that the high negative predictive value of the model is utilized to safely de-escalate care for low-risk patients while concentrating resources on those most likely to benefit from intervention. Additionally, to facilitate adoption in diverse settings, we envision the development of a lightweight web-based calculator or a mobile application that allows for real-time risk estimation and visualization of patient-level SHAP contributions, providing interpretable feedback that aligns with clinical reasoning.

Several limitations must be acknowledged to guide future research. First, the single-center retrospective design inherently restricts generalizability; despite rigorous internal validation, the model’s transportability across different populations and practice settings requires confirmation through multicenter external validation. Second, while multiple imputation and sensitivity analyses against complete-case analysis demonstrated the robustness of our findings, the assumption of “missing-at-random” for routine variables may not always hold true, and measurement variability (e.g., batch effects) could attenuate associations. Third, the model relies exclusively on static admission variables and does not incorporate dynamic clinical trajectories or imaging-derived biomarkers. Although this prioritizes model parsimony and deployability, it likely caps the maximum achievable predictive performance. Future work should focus on prospective validation and exploring the integration of time-series data to enhance predictive accuracy. Ultimately, transforming these optimized feature subsets into simplified scoring systems or nomograms could further bridge the gap between machine learning power and clinical usability, ensuring that the tool remains accessible at the bedside without requiring complex computational environments.

## Conclusion

This study demonstrates that a SHAP-guided random forest model can successfully predict atelectasis in MPP children using only the admission data. The model shows strong predictive performance and clinical interpretability, suggesting its potential value for early risk stratification. However, given the limitations of a single-center retrospective design, we emphasize that prospective multicenter validation is essential to confirm the model’s robustness and broader clinical applicability. Future studies involving diverse populations are warranted before this tool can be widely implemented in clinical practice.

## Supplementary Information

Supplementary Material 1: Supplement Figure 1. AUROC curves obtained using unimputed data in the training set. Note: Different colors represent different model algorithms

Supplementary Material 2: Supplement Figure 2. Calibration curve of the Random Forest model for predicting atelectasis. Note: Different colors represent different model algorithms.The dashed line represents perfect calibration (where predicted probabilities equal observed frequencies), while the solid blue line shows the performance of the calibrated Random Forest model. The histogram on the right side displays the distribution of sample counts across different predicted probability ranges. Model performance metrics are shown in the inset box: accuracy = 0.9649 and Brier score = 0.0234. A well-calibrated model would follow the diagonal dashed line, and the close alignment of the Random Forest curve with this line, particularly in the intermediate-to-high risk ranges, indicates good calibration performance

Supplementary Material 3: Supplement Figure 3. ROC curves and decision curve analysis of the LR model for predicting Mycoplasma pneumoniae pneumonia in children complicated by atelectasis. Note: Panel A depicts the ROC curve of the LR model in the training set; Panel B depicts the ROC curve in the testing set; Panel C illustrates the decision curve analysis for the training set (blue) and testing set (orange)

Supplementary Material 4: Supplement Table 1. Performance of Random Forest and Logistic Regression models on training and validation sets

## Data Availability

The datasets generated and/or analyzed during the current study are not publicly available due to institutional privacy policies but are available from the corresponding author on reasonable request.

## Abbreviations

MPP: Mycoplasma pneumoniae pneumonia
SHAP: SHapley Additive exPlanations
DT: Decision Tree
RF: Random Forest
ADB: Adaptive Boosting
XGB: Extreme Gradient Boosting
BDT: Bayesian Decision Tree
NDT: Neural Decision Tree
AUROC: Area Under the Receiver Operating Characteristic Curve
DCA: Decision Curve Analysis
PPV: Positive Predictive Value
NPV: Negative Predictive Value
WBC: White Blood Cell Count
NEU: Neutrophil Count
LYM: Lymphocyte Count
NEU%: Neutrophil Percentage
SAA: Serum Amyloid A
CRP: C-reactive Protein
hs-CRP: High-sensitivity C-reactive Protein
PLT: Platelet Count
CK-MB: Creatine Kinase-MB
LDH: Lactate Dehydrogenase
ALT: Alanine Aminotransferase
AST: Aspartate Aminotransferase
Ferr: Ferritin
IL-6: Interleukin-6
PCT: Procalcitonin
IgE: Immunoglobulin E
