# Supplementary figures and images for: SHAP based feature selection for machine learning prediction of mycoplasma pneumoniae pneumonia with atelectasis in children
Source: BMC Pediatr. 2026 Mar 9;26:328. doi: 10.1186/s12887-026-06647-3 (PMC13085413; doi:10.1186/s12887-026-06647-3)

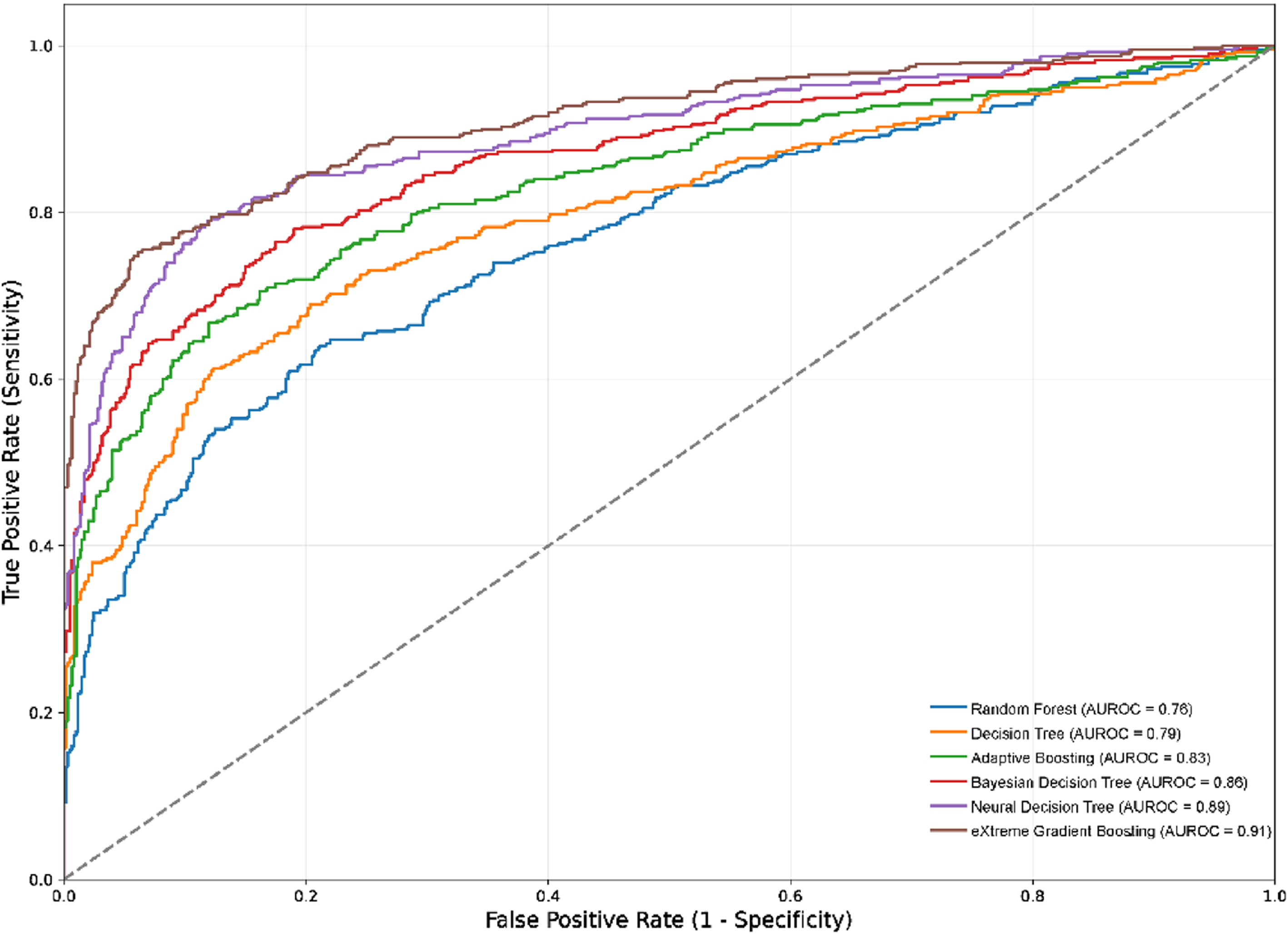

Supplement: Supplementary file 1 — Supplementary Material 1: Supplement Figure 1. AUROC curves obtained using unimputed data in the training set. Note: Different colors represent different model algorithms [file 12887_2026_6647_MOESM1_ESM.tif]

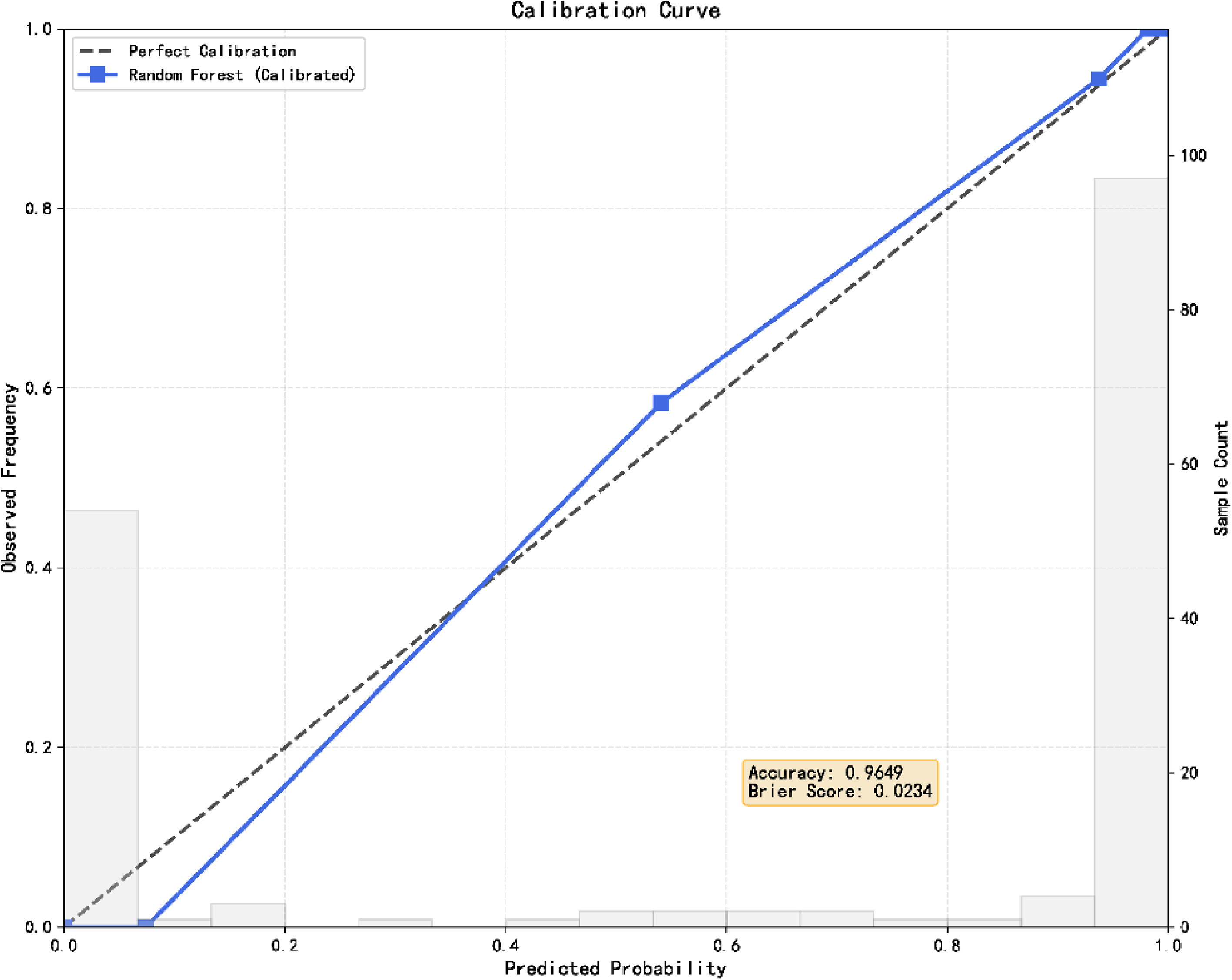

Supplement: Supplementary file 2 — Supplementary Material 2: Supplement Figure 2. Calibration curve of the Random Forest model for predicting atelectasis. Note: Different colors represent different model algorithms.The dashed line represents perfect calibration (where predicted probabilities equal observed frequencies), while the solid blue line shows the performance of the calibrated Random Forest model. The histogram on the right side displays the distribution of sample counts across different predicted probability ranges. Model performance metrics are shown in the inset box: accuracy = 0.9649 and Brier score = 0.0234. A well-calibrated model would follow the diagonal dashed line, and the close alignment of the Random Forest curve with this line, particularly in the intermediate-to-high risk ranges, indicates good calibration performance [file 12887_2026_6647_MOESM2_ESM.tif]

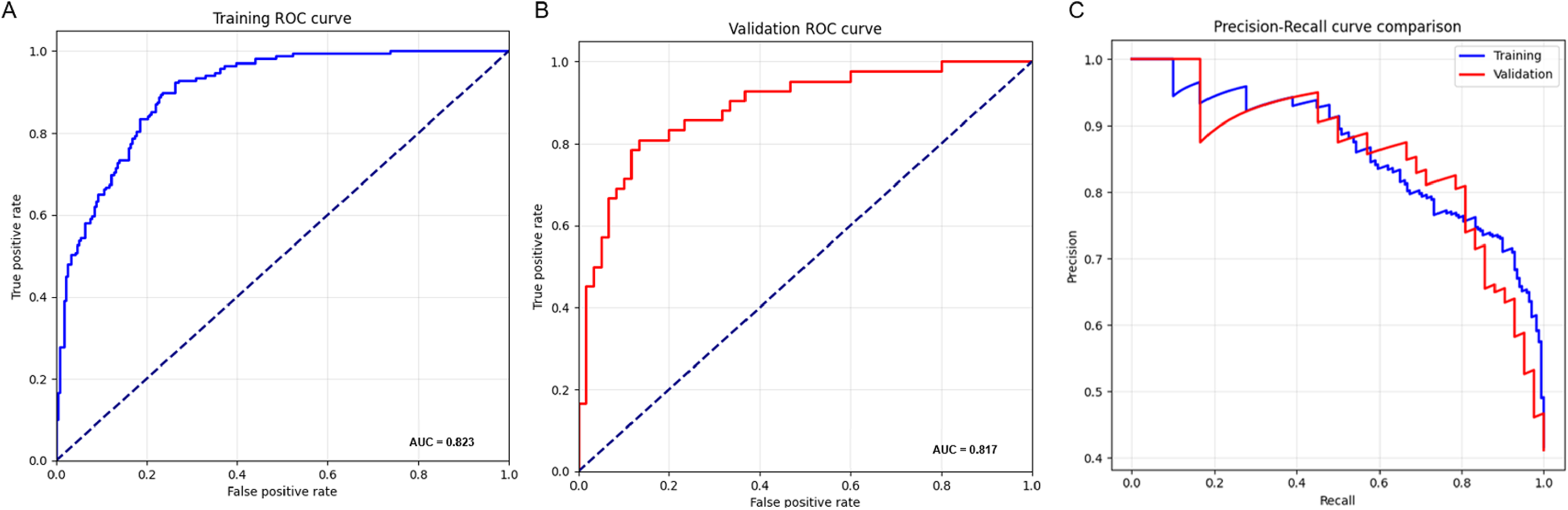

Supplement: Supplementary file 3 — Supplementary Material 3: Supplement Figure 3. ROC curves and decision curve analysis of the LR model for predicting Mycoplasma pneumoniae pneumonia in children complicated by atelectasis. Note: Panel A depicts the ROC curve of the LR model in the training set; Panel B depicts the ROC curve in the testing set; Panel C illustrates the decision curve analysis for the training set (blue) and testing set (orange) [file 12887_2026_6647_MOESM3_ESM.tif]
