# Supplementary material for: SHAP based feature selection for machine learning prediction of mycoplasma pneumoniae pneumonia with atelectasis in children
Source: BMC Pediatr. 2026 Mar 9;26:328. doi: 10.1186/s12887-026-06647-3 (PMC13085413; doi:10.1186/s12887-026-06647-3)
Supplement: Supplementary file 4 — Supplementary Material 4: Supplement Table 1. Performance of Random Forest and Logistic Regression models on training and validation sets [file 12887_2026_6647_MOESM4_ESM.docx]

Supplement Table 1. Performance of Random Forest and Logistic Regression models on training and validation sets

| Model | Accuracy | Precision / PPV | Recall / Sensitivity | F1 Score | AUROC | Specificity | NPV |
| --- | --- | --- | --- | --- | --- | --- | --- |
| Training | | | | | | | |
| LR | 0.82 | 0.79 | 0.70 | 0.74 | 0.87 | 0.87 | 0.80 |
| RF | 0.83 | 0.81 | 0.79 | 0.8 | 0.91 | 0.86 | 0.85 |
| Validation | | | | | | | |
| LR | 0.81 | 0.71 | 0.73 | 0.75 | 0.84 | 0.88 | 0.82 |
| RF | 0.81 | 0.77 | 0.79 | 0.78 | 0.89 | 0.83 | 0.85 |
